# Available tools to evaluate digital health literacy and engagement with eHealth resources: A scoping review

**DOI:** 10.1016/j.heliyon.2022.e10380

**Published:** 2022-08-23

**Authors:** Alice Faux-Nightingale, Fraser Philp, Darren Chadwick, Baldev Singh, Anand Pandyan

**Affiliations:** a Keele University, Newcastle under Lyme, UK; b University of Liverpool, Liverpool, UK; c University of Wolverhampton, Wolverhampton, UK; d Royal Wolverhampton Hospital, Wolverhampton, UK; e Bournemouth University, UK

**Keywords:** Access to care, Health literacy, Health technology/technology assessment, Information technology, Social deprivation

## Abstract

**Background:**

As eHealth and use of information and communication technologies (ICT) within healthcare becomes widespread, it is important to ensure that these forms of healthcare are accessible to the users. One factor that is key to accessing eHealth is digital health literacy.

**Objectives:**

This scoping review assesses available tools that can be used to evaluate digital health literacy.

**Methods:**

A systematic literature search was made in MEDLINE, CINAHL, APA PsychInfo, Ageline, AMED, and APA PsychArticles to present the tools currently in use to assess digital health literacy. A qualitative synthesis of the evidence was carried out using a data charting form created for this review. Extracted data included details of the population of investigation and digital health literacy tool used. A report was produced following PRISMA-ScR guidelines.

**Results:**

In total, 53 papers with adult participants and 3 with adolescent participants (aged between 12 and 19 years) were included in the scoping review. 5 questionnaires were identified that measured digital health literacy or attitudes towards the internet, of which the eHealth Literacy Scale (eHEALS) was the most commonly used questionnaire for both adults and children. Two children’s questionnaires were often accompanied by a second task to verify the accuracy of the responses to the eHEALS questions.

**Conclusions:**

eHEALS is the most commonly used method to assess digital health literacy and assess whether an individual is able to engage actively with eHealthcare or virtual resources. However, care needs to be taken to ensure that its administration does not exclude digitally disadvantaged groups from completing it. Future research would benefit from assessing whether digital health literacy tools are appropriate for use in clinical settings, working to ensure that any scales developed in this area are practical and can be used to support the allocation of resources to ensure that people are able to access healthcare equitably.

## 1 Introduction

The need to optimise healthcare services by drawing on technological solutions has long been recognised [1]. Recently there has been an increase in use of technology to disseminate information and services within the NHS, like an increase in telephone consultations for GPs [2], or using digital resources to disseminate information and support people. This provision has been accelerated during the recent Covid-19 pandemic and many of these facilities are likely to continue to be used beyond the pandemic [3]. While this shift increases the opportunities for people to access healthcare, it does not mean that they can access these resources equitably [4]. There are many barriers which can restrict user access to these resources [5]: infrastructure barriers (e.g. broadband provision, 4G available in their location etc.); financial and economic barriers, which can influence whether users have access to the necessary devices to engage with the resource (e.g. an internet-connected computer or phone that can access the resources); societal attitudes and exclusion, policy and governmental support; and education, training and individual support and impairment related barriers (e.g. skills to access and understand the information and language it is presented in) [1, 2]. If implementation of digital resources ignores these barriers, then there is a risk that existing divides in society associated with access and health may widen [6, 7].

Digital literacy is an essential skill that people require to engage with digital or online materials, it describes the ability to use the internet and other digital platforms and to find, understand, and evaluate the presented information [8]. Digital health literacy, or electronic health (eHealth) literacy, focuses on an individual’s ability to access, understand, and engage with digital healthcare materials or technology to contribute to quality of life [9]. As digital health resources become more widespread, it is important to assess individual’s ability to interact with these materials or technology to ensure that they are appropriate for the target audiences. By better understanding individual’s levels of digital health literacy, it is possible to identify the needs of specific groups to develop appropriate provision and ensure that the general public can access healthcare equitably.

This scoping review builds on existing research [9] to investigate the range of tools and techniques currently available to assess individual digital health literacy which can be utilised to inform the development of digital resources in the future. The aims are as follows: 1) present a narrative account of literature search findings and identify the methods used to assess digital health literacy, 2) identify and comment on the most used methods and any threshold criteria they may have to assess someone as digitally literate or not, in order to offer insight to guide the development of digital health facilities for the public in the future.

## 2 Methods

Literature searches were carried out in MEDLINE, CINAHL, APA PsychInfo, Ageline, AMED, and APA PsychArticles using EBSCO. The most recent search occurred on the 25/02/2021, no review protocol exists although the review followed the methodological framework described by Arksey and O’Malley [10] and the PRISMA-ScR [11] was used to guide the reporting of the findings. A copy of the PRISMA-ScR checklist is included as supplementary material.

The following search strategy was constructed to encompass techniques used to measure digital health literacy in healthcare: “(inclusion or divide or literacy or exclusion) AND (digital health or digital medicine or electronic health or ehealth or digital health care or mhealth) AND (questionnaire or survey or scale or instrument)”. The search strategy was applied to all fields, title, abstract, and full text. Papers identified in this search were filtered using the database search system to include papers from academic journals and remove duplicates. Papers not written in English were also filtered out of the search due to the associated cost and time of translation. A two stage screening process was conducted by two researchers. After reading the title and abstracts, one reviewer excluded articles which were not relevant to the review. Papers pertinent to the review question were separated into papers which included adult participants and those which investigated children and young people and these were sought for retrieval. Retrieved reports were read in full by two researchers to assess relevance for the study and to determine the methods used to investigate digital health literacy, with particular interest in the means of eliciting information. All eligible papers were included in the review (see Appendix 1 for the full list of included articles). Any key references identified in included papers' reference lists, for example where the paper referenced a separate questionnaire, were also searched for and included in this review.

A qualitative synthesis of the evidence was carried out using a data charting form created for this review. The authors recorded details of the publications included i.e. authors, year and location of the study before focusing on the aims of the study, the participant population and data collection method used to elicit information regarding participants' digital medical literacy. The questions used to collect information were recorded where possible. This data charting formed the basis of the analysis and was used to synthesise the results, no formal appraisal of evidence was required.

### 2.1 Patient and public involvement

Patients and the public were not involved in any way in this project.

## 3 Results

**Figure 1:**
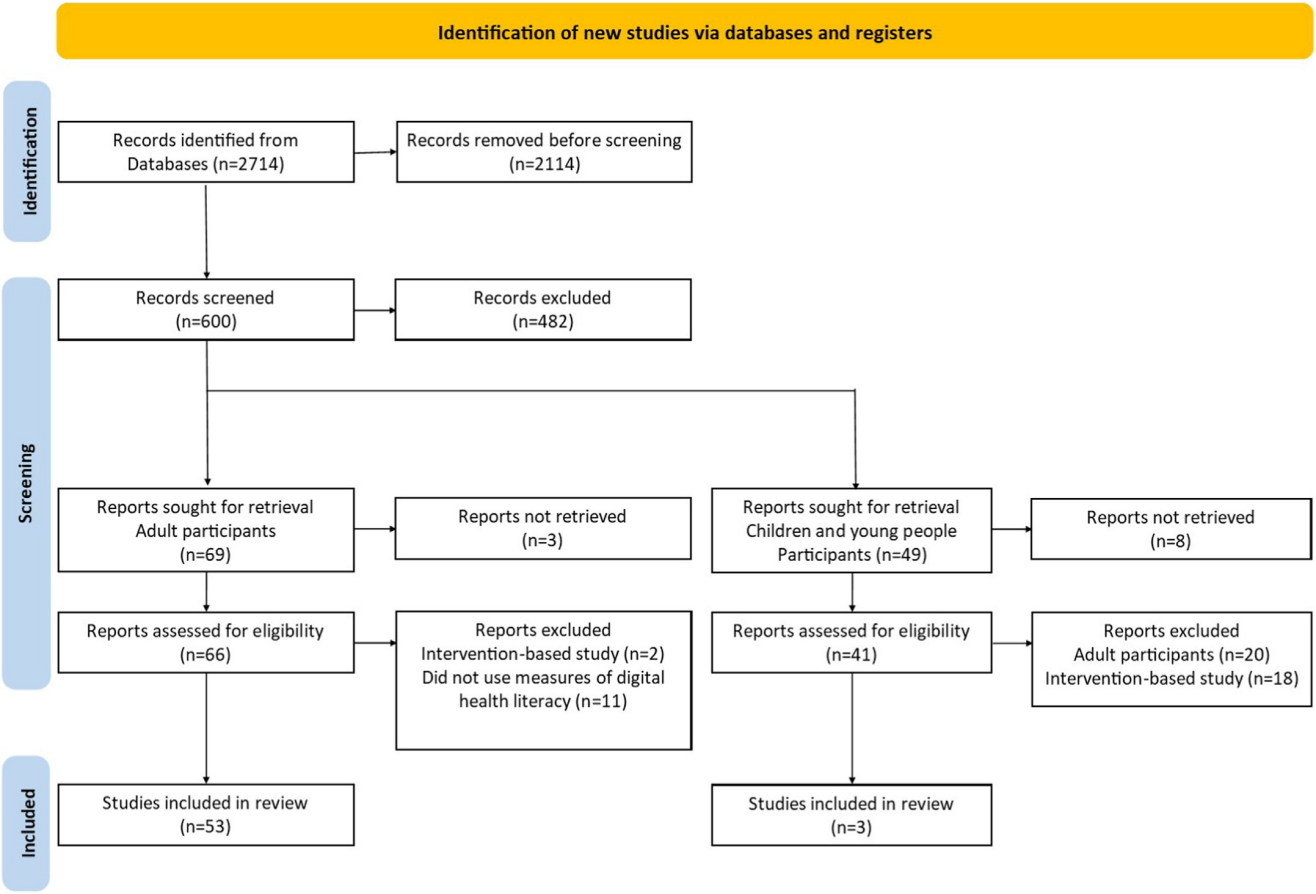
Flow chart of the paper selection process.

The database search identified 2714 papers of which 2595 were excluded following the process above, see Figure 1 for a flow chart of the selection process. After filtering for participant age, 66 papers were identified with adult participants. Of these, 13 were not relevant for the review as assessed by a full text read, either because they used technology or digital communication as an intervention rather than as a tool to evaluate literacy or engagement or because they did not include measures of digital health literacy. The literature search limited to younger participants produced 49 papers. Of these 20 featured adult participants rather than children, 18 used technology or digital communication as an intervention rather than as a tool to evaluate literacy or engagement, one referred to clinical telephone consultation for paediatric health care [12], while another used digital facilities to make literacy assessments [13].

In total, 53 papers with adult participants and 3 with adolescent participants (aged between 12 and 19 years) were included in the scoping review and were analysed to produce a narrative overview of the most used methods to assess digital health literacy.

### 3.1 Techniques used to elicit information from adults

A wide range of techniques were used to elicit information about digital health literacy. Most studies used surveys [14, 15, 16, 17, 18, 19, 20, 21, 22, 23, 24, 25, 26, 27, 28, 29, 30, 31, 32, 33, 34, 35, 36, 37, 38, 39, 40, 41, 42, 43, 44, 45, 46, 47, 48, 49] administered as online questionnaires [24, 26, 29, 41, 43, 45, 47], paper questionnaires [16, 17, 26, 38, 39, 40, 41, 50, 51], administered their survey via the telephone [17, 21, 40, 48], face-to-face [28, 35, 38, 42, 52], integrated into nursing practice/medical appointment [15, 25, 31, 53], or a combination of the above. Non-questionnaire techniques were less common but included the use of focus groups [54, 55] and semi-structured interviews [56, 57], which either investigated health literacy and perception of digital health resources through study specific question guides and wider discussion, or through the integration of the eHEALS questionnaire [54]; or the use of existing datasets [12, 52, 56, 58, 59, 60, 61, 62].

Five questionnaires were identified that measured digital health literacy: DHLI [16] (including a translated form of eHEALS), eHEALS [63], eHLA [29], EHLS [64], TeHLI [47], although a number of studies used surveys developed specifically for the project, some of which were based on the eHEALS questionnaire. See Table 1 for the populations that these surveys were used to investigate and further details about the measures.

**Table 1:** Details about the surveys used with adult participants which were identified within the scoping review.

Survey	Population (and Country) investigated	Frequency of use within papers in review	Areas of Digital Health/eHealth Literacy Assessed	N of Items	Scoring details	Psychometric properties of scales
Digital Health Literacy Instrument (DHLI)∗ [16]	General population (Netherlands) [16]	1	Internet use, health related internet use, health literacy, and eHealth literacy	21 questions	4 point Likert scale	Internal consistency, α = .87 (cronbach's alpha); Test-retest reliability, ICC = .77, p < .001
The eHealth Literacy Scale (eHEALS) [63]	Adults with COPD (US) [24], patients with recent fractures (Canada) [25], chronic patients (Ethiopia) [28], general population across generational groups (US) [23], Parents (Germany-Switzerland) [26], older adults (Canada; US and South Korea) [17, 18, 19, 25, 27], rural communities (Hawaii) [54], general population (Israel) [21], health professionals (Germany) [32], adult internet users (Japan) [34],	14	How to use internet and access resources, skills to evaluate health resources, confidence using internet resources	8 questions	5 point Likert scale	Internal consistency, α = .88 (cronbach's alpha). Test-retest reliability, scale score correlations ranged from r = .49- .68, intra-class correlation between different scores was .49. Additional papers have been published which assess reliability and validity of eHEALS in English and translated forms.
eHealth Literacy Assessment Toolkit (eHLA) [29]	General population and outpatient clinic (Denmark) [29]	1	Functional health literacy, health literacy, familiarity with health/care, disease knowledge, technology familiarity, technology confidence, incentives for engaging with technology	7 tools, 44 questions	Multiple-choice questions, and 4 point Likert scale	Internal consistency, Tool 1: α = .67, Tool 2: α = .85, Tool 3: α = .90, Tool 4: α = .59, Tool 5: α = .94, Tool 6: α = .91, Tool 7: α = .90 (cronbach's alpha).
eHealth Literacy Scale (eHLS) [64]	College students (Taiwan) [30]	1	Functional, interactive and critical eHealth literacy	12 questions	5 point Likert scale	Paper inaccessible due to language.
Transactional Model of eHealth Literacy (TeHLI) [47]	Patients [47]	1	Functional, communicative, critically analytical, and translational elements	18 questions	4 and 5 point Likert scale	Internal consistency, all scales: α = .87-.92 (cronbach’s alpha).

∗ integrated the eHEALS survey to measure eHealth literacy.

### 3.2 Psychometric properties of the scales

All questionnaires used ordinal based scoring criteria. These were predominantly Likert scales for measuring domains related to eHealth. Self-reported abilities regarding eHealth were reported as a spectrum rather than categories of overall ability. Although all available scales reported internal consistency ranging from α = .49–.92 (cronbach’s alpha), see Table 1 for further details, the included tools had limited validity in comprehensively measuring all domains required for navigating eHealth platforms and services. Psychometric properties of the included scales has previously been reported been elsewhere [65].

### 3.3 Threshold criteria for assessing digital literacy

Each of the measures was checked to see if it contained any threshold criteria which could be used to assess participants' eHealth literacy. All measures were developed as self-completed questionnaires, and none included specific assessments or tests of knowledge or skills. None of the measures included a threshold to determine an individual’s digital health literacy. Of the five, the eHLS was the only measure which resulted in an average score and discussed that score in relation to a ‘lower’ and ‘higher’ eHealth literacy scale. However, within the study the measure was not used in a clinical sense, and there was no threshold included which was used to determine whether an individual had a particular level of eHealth literacy. This study could not access the original measure paper and so is unable to state whether the scale was originally developed or used in a clinical setting or whether it has a defining threshold for eHealth literacy.

Of the scales identified in this scoping review, the eHealth Literacy Scale (eHEALS) [63] was the most commonly used measure, being used as an isolated scale in 13 studies and in combination with other scales in a further four. The eHEALS is a short questionnaire made up of 8 questions (Appendix 2). The scale was developed to determine individuals' abilities to access digital resources, and focus on perceived knowledge of how to use the internet to find resources, evaluate those resources for quality, and translate that knowledge into practice [63]. While originally tested on adolescents [63], the measure has been assessed for validity in a range of languages and was found to perform consistently across populations groups [19, 63, 66, 67, 68, 69, 70]. Papers in this review also described successfully using the eHEALS in a translated form [32, 34, 66, 71]. The papers which utilised the eHEALS investigated a wide range of populations in countries across the world, of which, older adults were the most commonly investigated group [17, 18, 19, 23, 25, 27].

### 3.4 Techniques used to elicit information from children and adolescents

Surveys were used to elicit information in all three papers investigating young populations (12–19 years). The eHEALS questionnaire was used in all three studies to assess digital health literacy, translated as appropriate [71, 72]. Two out of three papers incorporated additional techniques: Maitz and colleagues used a combination of eHEALS and researcher-led workshops where they asked children to navigate healthcare websites to gather information for a hypothetical scenario they had been presented with [72], they later looked at and scored the websites that the children had visited them to assess the accuracy of their eHEALS results. Ghaddar and colleagues [73] incorporated the Newest Vital Sign (NVS) [74] which measures general health literacy by asking a participant a series of comprehension questions to answer from information from an ice cream label.

## 4 Discussion

This scoping review presents an overview of the range of methods that have been used to investigate digital health literacy. Questionnaires were the most common method used to elicit information, with the eHEALS being the most commonly used questionnaire overall, translated as appropriate, and sometimes combined with other questions or methods as part of a broader investigation. The papers included in this review which investigated children and adolescents all used eHEALS to measure digital literacy, although secondary elements were often incorporated into data collection to gain a broader understanding of participants' health literacy.

As the NHS increases use of eHealth and digital healthcare resources, it is essential that care is taken to ensure target audiences can adequately access these facilities. The existing literature suggests that there are a wide range of factors which can act as barriers to populations accessing digital health [7], in particular: the skills to engage with the provision, and the technology and infrastructure to access it. One key limiting factor in enabling users to access digital health resources is digital health literacy. This scoping review identified that the eHEALS questionnaire is a commonly used measure to assess individuals' digital health literacy for health resources. The questions in the eHEALS questionnaire investigate a range of elements which make up digital health literacy, such as understanding and confidence using digital health resources and engaging with the presented information. This information provides insight of individuals' perceptions of how they interact information from digital health sources, and so it is likely that it could be a useful scale to determine individuals' digital health literacy or ability to access digital or eHealth resources.

The eHEALS questionnaire does not, however, provide a definitive assessment or score of digital health literacy which could be used to identify a population’s ability to engage with health resources in the digital world. Furthermore, as a self-reported measure its assessment qualities are limited by trusting the respondent to make an accurate assessment of their own abilities without any objective testing to confirm their judgement. It would therefore be inappropriate to use this as a means to accurately/objectively assess digital health literacy as a measure or standard of ability needed to engage with resources. This includes engagement without any accompaniment of further data collection features similar to those engaged by the adolescent studies described above [72, 73]. Future research would benefit from developing an objective questionnaire or platform which includes assessments of functional skills (not observed in any of the questionnaires utilised by the papers within this review). While the eHEALS can measure individuals' perceived ability to access information through the internet, a separate questionnaire or measure may be needed to determine individuals' ability to access online services in healthcare. Further questionnaires may also need to consider other factors, like attitudes toward digital provision of healthcare and information, social elements, or the user interface of the resources which may affect individuals' willingness to engage with digital health resources. For people with cognitive disabilities, it should also consider digital and health literacy supports which may be required. Furthermore, if intended for use in clinical practice, future questionnaires would benefit from a clear means to assess digital health literacy with a cut off point that can be used to categorise people according to their digital health literacy levels. This would inform health design and allocation of resources according to need, whether that is the development of new health materials or additional support for people with lower digital health literacy. The end goal is to allow people to access health services equitably, assessing digital health literacy and categorising people based on their capabilities ensures that interventions are accessible or that people are signposted to the best services for them.

Furthermore, though questionnaires are an effective means of eliciting information, care must be taken to ensure that the questionnaire and associated elements of research are equitably accessible and do not restrict participation. Administering the questionnaire using only digital means, for example, will exclude populations who lack either the access, skills or infrastructure to engage with the process - likely to also be those people who cannot engage with digital healthcare resources. Similarly, willingness to engage with the questionnaire will also impact on use and should be considered when developing new scales. Patient and clinician questionnaire fatigue should be considered when developing data elicitation methods, aiming to collect the maximum amount of information in the fewest number of questions. Likewise, any tools or questionnaires should be regularly revisited to ensure that they remain relevant and reflect updates to technology and digital services in what is a rapidly developing field.

With these considerations in mind, more accessible and adapted approaches to data collection may be necessary for gathering data, considering ease and speed of data collection for healthcare professionals, and accessibility for groups who may not be able to easily access the questionnaire, e.g. those with low literacy or cognitive impairments. Although some of the studies in this review attempted to mitigate these problems by the provision of paper copies of the questionnaire or through phone administration, this still does not make the research accessible to all populations and so risks misidentifying or overlooking existing divides in society.

### 4.1 Limitations

It is possible that this review may not have fully captured the range of methods used to investigate digital health literacy. Other studies may have used questionnaires, or similar, to investigate digital literacy but not incorporated the terms we used in our search strategy, or not investigated digital health literacy as a primary outcome measure. In these cases, the papers may not have appeared in our search findings and so will not have been included in the review. Since the screening process did not involve a second reviewer, the selection of the papers was based on a single perspective, and this may also have influenced the papers included in the review. While we acknowledge the limitations associated with this search strategy, given that our results were so conclusive, we feel that the impact of the exclusion of articles is minimal within this paper.

### 4.2 Conclusions

As health services increasingly incorporate the use of technology or digital based communications, it is essential that these modes of communication are considered as part of a wider appreciation of barriers to access digital healthcare. This review presented the range of surveys currently used to assess digital health literacy and which could be used to identify digital divides which may influence public access to healthcare. Out of the identified measures, the eHEALS questionnaire is the most commonly used, though it is limited in what it can assess. Future research would benefit from considering objective methods or ability thresholds to assess individuals' digital health literacy, or other factors which might influence public willingness/ability to engage with digital health services and resources. Identifying people who may struggle to access services makes it easier to better identify their healthcare needs and to guide resource allocation for further support to ensure that they are able to access health resources equitably. Since equity is fundamental in health care and inequity may be imparted by not addressing variation in digital ability, digital capital, digital literacy and more specifically, digital health literacy, it is critical to consider assessment tools, their ease of deployment and the utilisation of their outputs in practice as we further integrate digital health care delivery.

## Declarations

### Author contribution statement

All authors listed have significantly contributed to the development and the writing of this article.

### Funding statement

This research did not receive any specific grant from funding agencies in the public, commercial, or not-for-profit sectors.

### Data availability statement

No data was used for the research described in the article.

### Declaration of interest’s statement

The authors declare the following conflict of interests:

Professor Pandyan reports personal fees, unrestricted educational support, and non-financial support from Allergan, Biometrics Ltd., Ipsen, and Merz outside of the submitted work.

The other authors declare no competing interests.

### Additional information

Supplementary content related to this article has been published online at https://doi.org/10.1016/j.heliyon.2022.e10380.
