# Supplementary material for: Available tools to evaluate digital health literacy and engagement with eHealth resources: A scoping review
Source: Heliyon. 2022 Aug 23;8(8):e10380. doi: 10.1016/j.heliyon.2022.e10380 (PMC9449566; doi:10.1016/j.heliyon.2022.e10380)
Supplement: Multimedia component 2 [file mmc2.docx]

**APPENDICES**

**Appendix 1 - References for included papers in adults review**

| Alvarez-Galvez, J., et al. (2020). "The persistence of digital divides in the use of health information: a comparative study in 28 European countries." International Journal of Public Health 65(3): 325-333. |
| --- |
| Asan, O., et al. (2018). "Preferences for Health Information Technologies Among US Adults: Analysis of the Health Information National Trends Survey." Journal of Medical Internet Research 20(10): 72-72. |
| Athanasopoulou, C., et al. (2017). "Internet use, eHealth literacy and attitudes toward computer/internet among people with schizophrenia spectrum disorders: a cross-sectional study in two distant European regions." BMC Medical Informatics & Decision Making 17: 1-14. |
| Chan, C. V. and D. R. Kaufman (2011). "A framework for characterizing eHealth literacy demands and barriers." Journal of Medical Internet Research 13(4): e94-e94. |
| Cherid, C., et al. (2020). "Current level of technology use, health and eHealth literacy in older Canadians with a recent fracture—a survey in orthopedic clinics." Osteoporosis International 31(7): 1333-1340. |
| Choi, N. G. and D. M. Dinitto (2013). "The Digital Divide Among Low-Income Homebound Older Adults: Internet Use Patterns, eHealth Literacy, and Attitudes Toward Computer/Internet Use." Journal of Medical Internet Research 15(5): e93-e93. |
| Cottrell, M. A., et al. (2018). "Patients are willing to use telehealth for the multidisciplinary management of chronic musculoskeletal conditions: A cross-sectional survey." Journal of Telemedicine & Telecare 24(7): 445-452. |
| de Guzman, A. B. and M. J. S. Diño (2020). "Examining the role of Filipino elderly attitudes toward computer and internet on their behavioral intention for telehealth participation." Educational Gerontology 46(3): 140-149. |
| Del Giudice, P., et al. (2018). "Correlation Between eHealth Literacy and Health Literacy Using the eHealth Literacy Scale and Real-Life Experiences in the Health Sector as a Proxy Measure of Functional Health Literacy: Cross-Sectional Web-Based Survey." Journal of Medical Internet Research 20(10): 53-53. |
| Ghaddar, S., et al. (2020). "Understanding the Intention to Use Telehealth Services in Underserved Hispanic Border Communities: Cross-Sectional Study." Journal of Medical Internet Research 22(9): N.PAG-N.PAG. |
| Hall, A. K., et al. (2015). "The Digital Health Divide: Evaluating Online Health Information Access and Use Among Older Adults." Health Education & Behavior 42(2): 202-209. |
| Hayat, T., et al. (2017). "With Some Help From My Network: Supplementing eHealth Literacy With Social Ties." Journal of Medical Internet Research 19(3): 1-1. |
| Heayon, L., et al. (2020). "Discrepancies in Demand of Internet of Things Services Among Older People and People With Disabilities, Their Caregivers, and Health Care Providers: Face-to-Face Survey Study." Journal of Medical Internet Research 22(4): 1-16. |
| Hennemann, S., et al. (2017). "Ready for eHealth? Health Professionals' Acceptance and Adoption of eHealth Interventions in Inpatient Routine Care." Journal of Health Communication 22(3): 274-284. |
| Juvalta, S., et al. (2020). "Electronic Health Literacy in Swiss-German Parents: Cross-Sectional Study of eHealth Literacy Scale Unidimensionality." Journal of Medical Internet Research 22(3): N.PAG-N.PAG. |
| Karnoe, A., et al. (2018). "Assessing Competencies Needed to Engage With Digital Health Services: Development of the eHealth Literacy Assessment Toolkit." Journal of Medical Internet Research 20(5): 1-1. |
| Kasparian, N. A., et al. (2017). "eHealth literacy and preferences for eHealth resources in parents of children with complex CHD." Cardiology in the Young 27(4): 722-730. |
| Khairat, S., et al. (2019). "Would Geriatric Patients Accept Using a Telemedicine Platform for Post ICU-Discharge Follow-Up Visits?...MEDINFO 2019, the 17th World Congress on Medical and Health Informatics, August 25-30, 2019, Lyon, France." Studies in Health Technology & Informatics 264: 1233-1237. |
| Knudsen, M. V., et al. (2020). "Tele-rehabilitation and hospital-based cardiac rehabilitation are comparable in increasing patient activation and health literacy: A pilot study." European Journal of Cardiovascular Nursing 19(5): 376-385. |
| Kontos, E., Blake, K. D., Chou, W. Y. S., & Prestin, A. (2014). Predictors of eHealth usage: insights on the digital divide from the Health Information National Trends Survey 2012. Journal of medical Internet research, 16(7), e172. |
| Kuek, A. and S. Hakkennes (2020). "Healthcare staff digital literacy levels and their attitudes towards information systems." Health Informatics Journal 26(1): 592-612. |
| Kurtin, S. W. (2018). "Mobile Health, Technology Engagement and Communicative Health Literacy in Older Adult Cancer Survivors." Mobile Health, Technology Engagement & Communicative Health Literacy in Older Adult Cancer Survivors: 1-1. |
| Lee, D. R., et al. (2021). Understanding the Uptake of Digital Technologies for Health‐Related Purposes in Frail Older Adults. Malden, Massachusetts, Wiley-Blackwell. 69: 269-272. |
| Lee, O. E.-K., et al. (2020). "Factors affecting information and communication technology use and eHealth literacy among older adults in the US and South Korea." Educational Gerontology 46(9): 575-586. |
| Manafò, E. and S. Wong (2013). "eSEARCH©: A Tool to Promote the eHealth Literacy Skills of Older Adults." Journal of Consumer Health on the Internet 17(3): 255-271. |
| Melholt, C., et al. (2018). "Cardiac patients' experiences with a telerehabilitation web portal: Implications for eHealth literacy." Patient Education & Counseling 101(5): 854-861. |
| Mitsutake, S., et al. (2016). "Associations of eHealth Literacy With Health Behavior Among Adult Internet Users." Journal of Medical Internet Research 18(7): 5-5. |
| Paige, S. R., et al. (2018). "Electronic Health Literacy Across the Lifespan: Measurement Invariance Study." Journal of Medical Internet Research 20(7): 266-279. |
| Paige, S. R., et al. (2019). "Transactional eHealth Literacy: Developing and Testing a Multi-Dimensional Instrument." Journal of Health Communication 24(10): 737-748. |
| Park, H., et al. (2016). "Identifying Health Consumers' eHealth Literacy to Decrease Disparities in Accessing eHealth Information." CIN: Computers, Informatics, Nursing 34(2): 71-99. |
| Percival, J. and J. Hanson (2006). "Big brother or brave new world? Telecare and its implications for older people's independence and social inclusion." Critical Social Policy 26(4): 888-909. |
| Poli, A., et al. (2019). "A research tool for measuring non-participation of older people in research on digital health." BMC Public Health 19(1): 1-12. |
| Powell, J. and U. Deetjen (2019). "Characterizing the Digital Health Citizen: Mixed-Methods Study Deriving a New Typology." Journal of Medical Internet Research 21(3): N.PAG-N.PAG. |
| Sand-Jecklin, K., et al. (2017). "Incorporating Health Literacy Screening Into Patients' Health Assessment." Clinical Nursing Research 26(2): 176-190. |
| Schickedanz, A., et al. (2013). "Access, interest, and attitudes toward electronic communication for health care among patients in the medical safety net." JGIM: Journal of General Internal Medicine 28(7): 914-920. |
| Seon-Yoon, C. and N. Eun-Shim (2015). "Testing Reliability and Validity of the eHealth Literacy Scale (eHEALS) for Older Adults Recruited Online." CIN: Computers, Informatics, Nursing 33(4): 150-156. |
| Serafica, R., et al. (2019). "The Use of Mobile Health to Assist Self-management and Access to Services in a Rural Community." CIN: Computers, Informatics, Nursing 37(2): 62-72. |
| Shiferaw, K. B., et al. (2020). "E-health literacy and associated factors among chronic patients in a low-income country: a cross-sectional survey." BMC Medical Informatics & Decision Making 20(1): 1-9. |
| Shu-Ching, Y., et al. (2017). "The Associations Among Individual Factors, eHealth Literacy, and Health-Promoting Lifestyles Among College Students." Journal of Medical Internet Research 19(1): 1-10. |
| Sin, D. Y. E., et al. (2020). "Assessment of willingness to Tele-monitoring interventions in patients with type 2 diabetes and/or hypertension in the public primary healthcare setting." BMC Medical Informatics & Decision Making 20(1): 1-11. |
| Stellefson, M., et al. (2019). "Association Between Health Literacy, Electronic Health Literacy, Disease-Specific Knowledge, and Health-Related Quality of Life Among Adults With Chronic Obstructive Pulmonary Disease: Cross-Sectional Study." Journal of Medical Internet Research 21(6): N.PAG-N.PAG. |
| Theodoulou, E., et al. (2019). "Preferences and attitudes to mobile phone and Internet‐based cardiac rehabilitation maintenance programs in rural Australia." Australian Journal of Rural Health 27(2): 179-180. |
| Tyagi, S., et al. (2018). "Acceptance of Tele-Rehabilitation by Stroke Patients: Perceived Barriers and Facilitators." Archives of Physical Medicine & Rehabilitation 99(12): 2472-2472. |
| van der Vaart, R. and C. Drossaert (2017). "Development of the Digital Health Literacy Instrument: Measuring a Broad Spectrum of Health 1.0 and Health 2.0 Skills." Journal of Medical Internet Research 19(1): 1-12. |
| van Houwelingen, C. T. M., et al. (2018). "Understanding Older People's Readiness for Receiving Telehealth: Mixed-Method Study." Journal of Medical Internet Research 20(4): 1-1. |
| Vollbrecht, H., et al. (2020). "Evaluating the Need to Address Digital Literacy Among Hospitalized Patients: Cross-Sectional Observational Study." Journal of Medical Internet Research 22(6): N.PAG-N.PAG. |
| Wallston, K. A., et al. (2014). "Psychometric properties of the brief health literacy screen in clinical practice." JGIM: Journal of General Internal Medicine 29(1): 119-126. |
| Warring, C. D., et al. (2018). "Implementation of a Routine Health Literacy Assessment at an Academic Medical Center." Journal for Healthcare Quality: Promoting Excellence in Healthcare 40(5): 247-255. |
| Zheng, J. and H. Yu (2019). "QuikLitE, a Framework for Quick Literacy Evaluation in Medicine: Development and Validation." Journal of Medical Internet Research 21(2): N.PAG-N.PAG. |

**References for included papers in children’s review**

Gazibara, T., et al. (2020). "Searching for online health information instead of seeing a physician: a cross-sectional study among high school students in Belgrade, Serbia." International Journal of Public Health 65(8): 1269-1278.

Ghaddar, S. F., et al. (2012). "Adolescent health literacy: the importance of credible sources for online health information." The Journal of school health 82(1): 28-36.

Maitz, E., et al. (2020). "Internet-Based Health Information-Seeking Behavior of Students Aged 12 to 14 Years: Mixed Methods Study." Journal of Medical Internet Research 22(5): e16281.

**Appendix 2: eHEALS – eHealth Literacy Scale (Norman & Skinner, 2006)**

**I would like to ask you for your opinion and about your experience using the Internet for health information. For each statement, tell me which response best reflects your opinion and experience *right now*.**

1. I know **what** health resources are available on the Internet

1) Strongly Disagree

2) Disagree

3) Undecided

4) Agree

5) Strongly Agree

2. I know **where** to find helpful health resources on the Internet

1) Strongly Disagree

2) Disagree

3) Undecided

4) Agree

5) Strongly Agree

3. I know **how** to find helpful health resources on the Internet

1) Strongly Disagree

2) Disagree

3) Undecided

4) Agree

5) Strongly Agree

4. I know **how to use** the Internet to answer my questions about health

1) Strongly Disagree

2) Disagree

3) Undecided

4) Agree

5) Strongly Agree

5. I know how to use **the health information** I find on the Internet to help me

1) Strongly Disagree

2) Disagree

3) Undecided

4) Agree

5) Strongly Agree

6. I have the skills I need to **evaluate** the health resources I find on the Internet

1) Strongly Disagree

2) Disagree

3) Undecided

4) Agree

5) Strongly Agree

7. I can tell **high quality** health resources from **low quality** health resources on the Internet

1) Strongly Disagree

2) Disagree

3) Undecided

4) Agree

5) Strongly Agree

8. I feel **confident** in using information from the Internet to make health decisions

1) Strongly Disagree

2) Disagree

3) Undecided

4) Agree

5) Strongly Agree

***Thank you!***
